# Bleeding Diathesis in Multiple Myeloma: A Rare Presentation of a Dreadful Emergency With Management Nightmare

**DOI:** 10.7759/cureus.13990

**Published:** 2021-03-19

**Authors:** Sabih Rahman, Sindhusha Veeraballi, Kok Hoe Chan, Hamid S Shaaban

**Affiliations:** 1 Internal Medicine, Saint Michael's Medical Center, Newark, USA; 2 Medical Education, Saint Michael's Medical Center, Newark, USA; 3 Hematology and Oncology, Saint Michael's Medical Center, Newark, USA

**Keywords:** bleeding diathesis, multiple myeloma, acquired dysfibrinogenemia

## Abstract

Multiple myeloma is a neoplastic disorder of plasma cells. An abnormal coagulation profile, though commonly seen in multiple myeloma, can rarely manifest as life-threatening hemorrhagic complications. Bleeding tendencies in multiple myeloma can be explained by a variety of mechanisms such as dysfibrinogenemia, paraprotein-induced platelet dysfunction, shortened platelet survival, damage to the vascular endothelium, and acquired von-Willebrand syndrome. Herein, we report a 61-year-old female who presented with the signs and symptoms of hemorrhagic shock with multiple myeloma, which remained refractory to a massive transfusion protocol. Her condition stabilized when she was started on dexamethasone and antifibrinolytic infusion targeting acquired dysfibrinogenemia. To the best of our knowledge, hemorrhagic shock secondary to dysfibrinogenemia is an unusual phenomenon in multiple myeloma.

## Introduction

Multiple myeloma is plasma cell dyscrasias with the presence of excess monoclonal paraprotein resulting from the abnormal proliferation of monoclonal plasma cells in the bone marrow [1]. Multiple myeloma was first described in 1848, represents 1% of all malignancies worldwide, more than 10% of all hematological malignancies, and is the 14th most common cause of cancer deaths in the United States [2-3]. Various somatic and oncogenic mutations and chromosomal aberrations are associated with the mutation of plasma cells, which leads to the formation of a premalignant condition called monoclonal gammopathy of undetermined significance (MGUS) that then progresses to smoldering multiple myeloma. Further progression develops symptomatic and fatal multiple myeloma [2].

An abnormal coagulation profile, though commonly seen in multiple myeloma, can rarely manifest as life-threatening hemorrhagic complications. Bleeding tendencies in multiple myeloma can be explained by a variety of mechanisms such as dysfibrinogenemia secondary to the inhibition of fibrin monomers by the FAB portion of paraprotein molecules, paraprotein-induced platelet dysfunction, shortened platelet survival, damage to vascular endothelium, and acquired von-Willebrand syndrome [4].

Herein, we report a 61-year-old female who presented with signs and symptoms of hemorrhagic shock. She remained refractory to a massive transfusion protocol with packed red blood cells, platelets, coagulation factor replacement, vitamin K, folic acid therapy, and desmopressin. Her condition stabilized when she was started on dexamethasone and antifibrinolytic infusion targeting acquired dysfibrinogenemia. Later, she was diagnosed with multiple myeloma and remarkably responded to the treatment of multiple myeloma. Hemorrhagic shock secondary to dysfibrinogenemia is an unusual phenomenon in multiple myeloma.

## Case presentation

**Figure 1:**
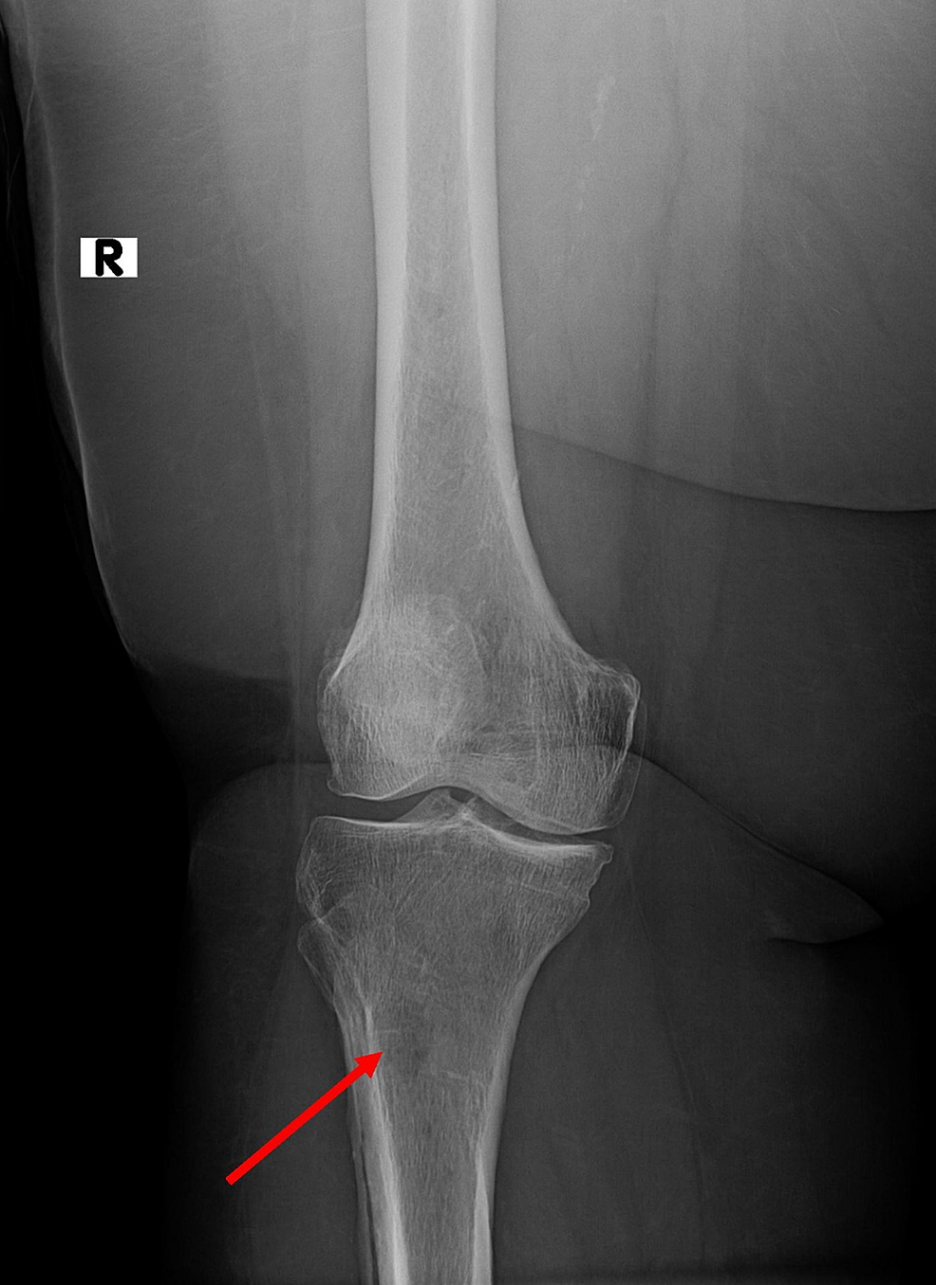
Skeletal survey showed lytic lesions of her long bone shafts

A 61-year-old female with a past medical history of hypertension and coronary artery disease status post-percutaneous coronary intervention presented to the emergency room with generalized weakness and dizziness for a day. On initial encounter, her temperature was 99.8 degrees Fahrenheit, respiratory rate 18 breaths/min, heart rate 115 bpm, and blood pressure 77/33 mmHg. Her initial laboratory data revealed hemoglobin of 5.5 g/dL (13.5-17.5 g/dL), which dropped to 3.6 g/dL in six hours, platelets of 142k (150k-450k). Coagulation studies revealed fibrin degradation products <5 mg/dL (<10 mg/L), low haptoglobin 13.7 mg/dL (50-220 mg/dL), low fibrinogen 156 mg/dL (200-400 mg/dL), prolonged prothrombin time 22.1s (11-12.5s), international normalized ratio (INR) 1.82 (<1.1), and prolonged partial thromboplastin time 58.7s (30-40s). Factor VIII activity was 184% (57%-163%) and von Willebrand factor (vWF) activity was normal 150% (50%-200%). Direct Coombs test was negative, and reduced activity of coagulation factors of II, VII, and X activity was found. Blood urea nitrogen was 56 mg/dL (6-24 mg/dL) and creatinine was 3.5 mg/dL (0.5-1.0 mg/dL). The septic workup was negative. She was transferred to the medical intensive care unit where she remained unstable even after immediate fluid resuscitation and massive transfusion protocol. She received vitamin K, vitamin B12, folic acid, desmopressin to address uremic platelet dysfunction, antifibrinolytic (Amicar) infusion, and intravenous dexamethasone to address acquired dysfibrinogenemia after which her bleeding finally stopped. She was found to have an M-spike (61.8%, 33.4 mg/dL) on protein electrophoresis, hypercalcemia, and lytic lesions of her long bone shafts on the skeletal survey (Figure 1).

**Figure 2:**
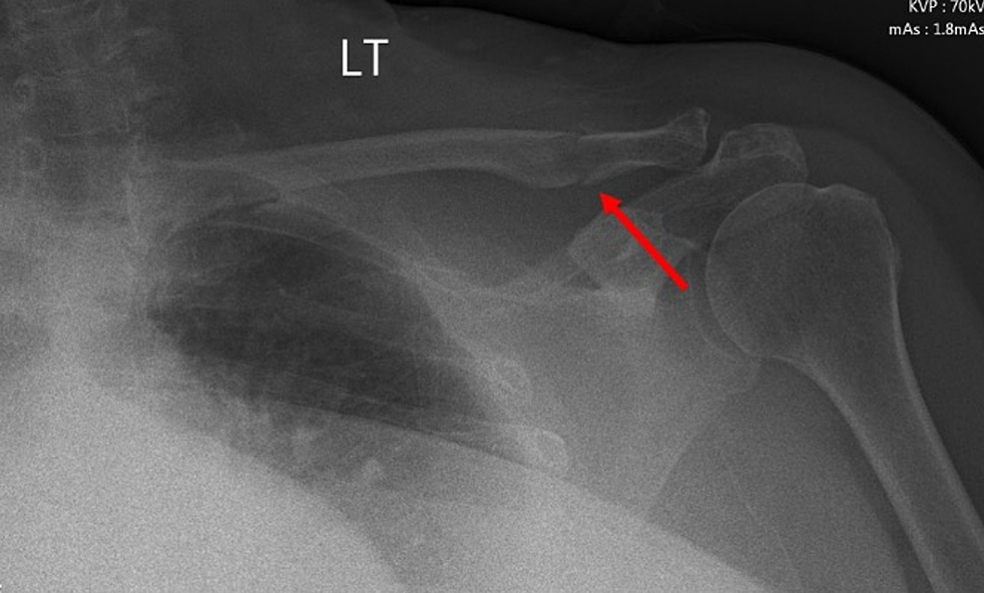
Chest X-ray revealed a non-displaced fracture of her left clavicle

Her chest X-ray revealed a non-displaced fracture of her left clavicle (Figure 2).

**Figure 3:**
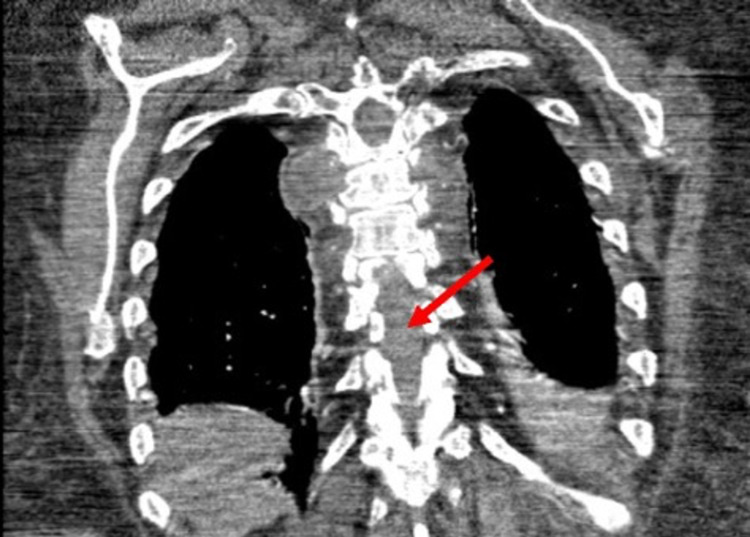
The CT scan showed a paravertebral soft tissue mass at the T6-T7 level with the destruction of part of the T6 vertebral body

A new CT scan showed subtle lytic lesions involving the anterior aspect of her second left rib. It also showed paravertebral soft tissue mass at the T6-T7 level with the destruction of part of the T6 vertebral body (Figure 3).

Further immunohistochemical analysis of the bone marrow biopsy showed 60%-70% of CD138 positive and lambda light chains expressing monoclonal lambda/immunoglobulin G (IgG) plasma cells confirming the diagnosis of lambda/IgG multiple myeloma. The coagulopathy was controlled when she was started on chemotherapy for multiple myeloma. On follow-up, her fibrinogen was remarkedly improved to 244 mg/dl (200-400 mg/dl).

## Discussion

Multiple myeloma commonly presents with a wide variety of symptoms, known as CRAB symptoms, with C - hypercalcemia (13%), R - renal failure (20%-40%), A - anemia (70%), and B - lytic bone lesions (80%), in addition to weight loss (24%) and recurrent infections [4]. The National Comprehensive Cancer Network (NCCN) criteria for the diagnosis of active multiple myeloma includes clonal bone marrow plasma cells ≥10%, the presence of biopsy-confirmed bony or extramedullary plasmacytoma, and one or more myeloma-defining events such as hypercalcemia (>1 mg/dl higher than the upper limit), renal failure (creatinine > 2 mg/dL or creatinine clearance < 40 mL/min), anemia (hemoglobin < 10 g/dL or hemoglobin > 2 g/dL below the lower limit of normal), one or more osteolytic bone lesions on skeletal radiography, one or more focal ≥5 mm lesions on MRI scans, clonal bone marrow plasma cells ≥60%, abnormal serum-free light chain (FLC) ratio ≥100 (involved kappa) or < 0.01 (involved lambda) [5].

The malignant plasma cells produce monoclonal paraprotein, also called M protein, which can bind to any coagulation factor, platelet, von-Willebrand factor, or fibrinogen and can thus cause diverse hemostatic abnormalities [6-7]. Abnormal coagulation tests are commonly seen in plasma cell dyscrasias. However, clinically significant bleeding complications are rarely seen. Several retrospective studies on multiple myeloma did not report bleeding as a presenting symptom [8]. Bleeding rates of 13%, 33%, and 36% are seen with IgG, IgA, IgM M proteins, respectively [7] and were reported in a study on 62 patients by Perkins [9]. Due to the limited experience, there is a scarcity of literature available on the management of bleeding complications in plasma cell dyscrasias.

Bleeding tendencies in multiple myeloma can be explained by a variety of mechanisms such as dysfibrinogenemia secondary to the inhibition of fibrin monomers by the FAB portion of paraprotein molecules, paraprotein-induced platelet dysfunction, shortened platelet survival, damage to vascular endothelium, and acquired von-Willebrand syndrome [3]. In our patient, the pathology behind bleeding diathesis is thought to be acquired dysfibrinogenemia with the evidence of prolonged prothrombin time and decreased fibrinogen activity. The patient had the normal activity of von-Willebrand factor, Factor VIII, and IX activity, which ruled out acquired hemophilia in this context.

Treatment therapies that can be used for the symptomatic management of bleeding complications include coagulation factor replacement, fibrinolysis inhibitors, protamine sulfate, Arginine, vasopressin, and platelet factor 4. Plasma exchange and splenectomy can be tried in refractory cases [8]. Though symptomatic treatment can improve the patient's condition temporarily, the mainstay of treatment is always treating the underlying disease. Our patient received symptomatic treatment, including massive transfusion protocol with packed red blood cells, platelets, coagulation factor replacement, vitamin K, and folic acid therapy. Her condition temporarily stabilized with the transfusion of antifibrinolytic (Amicar) targeting acquired dysfibrinogenemia. However, coagulopathy was controlled only when the patient was started on chemotherapy for multiple myeloma.

## Conclusions

Multiple myeloma can rarely present with bleeding complications, which, when untreated, may progress to hemorrhagic shock. It is paramount to have a high index of suspicion for multiple myeloma in a patient with acquired bleeding disorders who is refractory to treatment. Coagulopathy can be controlled only when the patient receives treatment for their underlying disease.
